# Bivalent Fears of Evaluation in Social Anxiety: Evaluation of an Extended Psychoevolutionary Model

**DOI:** 10.3390/ejihpe14110191

**Published:** 2024-11-12

**Authors:** Glen W. Bates, Pragalathan Apputhurai, Simon R. Knowles

**Affiliations:** 1 Department of Psychology, School of Health Sciences, Swinburne University of Technology, Melbourne, VIC 3122, Australia; sknowles@swin.edu.au; 2 Department of Health Sciences and Biostatistics, School of Health Sciences, Swinburne University of Technology, Melbourne, VIC 3122, Australia; papputhurai@swin.edu.au

**Keywords:** social anxiety disorder, fear of negative evaluation, fear of positive evaluation, bivalent fear of evaluation model

## Abstract

Fears of negative evaluation (FNEs) and fears of positive evaluation (FPEs) comprise a bivalent model of evaluation that can explain the aetiology and maintenance of Social Anxiety Disorder (SAD). In this study, we examined an extended version of this model which incorporates two related cognitive processes (concerns about reprisal and discounting of positive outcomes) as partial mediators of the effects of FNEs and FPEs. We built on earlier work by including a broader measure of social anxiety across different social situations and comparing models for groups of participants with and without probable SAD. Structural equation modelling was utilised to test the model in a sample of 890 university students (74.8% female, mean age 29.49). We replicated the findings of Cook et al. in the overall sample and in the group with probable SAD. FNEs and FPEs predicted social anxiety directly and were serially mediated by concerns about reprisal and discounting positive outcomes. The model was also a good fit for those without SAD; however, in the model, FNEs were no longer a direct predictor of social anxiety. The findings confirm the utility of the extended bivalent model and have implications for psychoevolutionary accounts of social anxiety.

## 1. Introduction

Social anxiety is endemic within interpersonal interactions and the level of impairment it causes rests on a continuum of distress [1,2]. At the lowest end of the continuum is the mild apprehension generated by social situations involving direct scrutiny by others (e.g., public speaking, job interviews) or informal social interactions that include possible evaluation by others (e.g., parties, dating). At the upper end is the marked impairment in social and occupational functioning and crippling anxiety experienced by people who meet criteria for Social Anxiety Disorder (SAD). Consistent with the ubiquity of social anxiety in daily life, SAD is the most common of the anxiety disorders and is one of the most common psychiatric conditions in communities worldwide [3,4].

According to the Technical Revision of the Fifth Edition of The Diagnostic and Statistical Manual of Mental Disorders (DSM-5-TR, American Psychiatric Association [APA] [5]), the essential features of SAD are intense anxiety and fear of social situations involving scrutiny by others that can elicit negative evaluation. These intense fears of negative evaluation are nearly always present in social situations the person finds difficult and are out of proportion to the actual level of social risk. Recognition that the fear of negative evaluation (FNE) is central to SAD was introduced in DSM-5 [6]. This broadened the focus of diagnosis beyond immediate feelings of embarrassment or humiliation to encompass other concerns such as fear of offending others or fear of rejection [7]. The emphasis on FNE also aligned the DSM system with cognitive behavioural conceptions of SAD which provide the most established theoretical explanations and effective treatments of social anxiety [8,9].

Cognitive–behavioural models of social anxiety (e.g., [10,11]) view FNE as a core cognitive factor in the disorder and an important target in treatment because it drives worry and distress in social situations. FNE emanates from a negative view of self [12]. People with problematic social anxiety hold fixed views of themselves as defective and flawed [8], and these views are associated with intense self-dissatisfaction and self-criticism [13]. During social interactions, this focuses attention on the reactions of others and biases interpretation of those reactions towards critical appraisals. Such perceived negative evaluations are distressing and are taken as confirmation of the negative view of self which in turn activates concerns about rejection and social exclusion [14].

In this study, we sought to build on previous research that has established that fears of evaluation in social anxiety are not restricted to negative evaluations but extend to all forms of evaluation, including positive evaluation, and contribute to a bivalent model of social anxiety [1]. We also explored two related cognitive processes found to be important in determining the influence of fears of evaluation. These include the level of fears of social reprisal by others who form evaluations of individuals and the tendency of socially anxious people to discount their positive performances and emphasise failures [15]. Another way in which we built on past research was by providing a broader measure of social anxiety concerns than has previously been used in tests of the bivalent model. Our measure of social anxiety incorporated different types of situations that elicit social anxiety, including performance-related anxiety (e.g., job interviews) and informal interactions (e.g., parties). As a fourth extension of previous work, we directly compared a model of evaluative concerns in people with probable SAD to a group of people without SAD.

### 1.1. The Bivalent Fear of Evaluation Model of Social Anxiety

Research suggests that fears of the personal consequences of judgments of the self by others are not confined to negative evaluations. Almost paradoxically, socially anxious people have also been found to fear positive evaluations. Weeks and colleagues (e.g., [16,17,18]) have shown that socially anxious people show intense apprehension and distress after direct favourable social comparisons with others in public situations. The fear of positive evaluation (FPE) produces anxiety as such public evaluations place the person “in the spotlight” and result in them feeling conspicuous (Weeks [19]). Weeks et al. [16] concluded that the presence of both positive and negative fears of evaluation suggests that the fear is of evaluation in general and combined these social anxiety-related fears into a bivalent fear of evaluation model (BFOE) of social anxiety.

The dual processes of FNE and FPE in social anxiety have been explained from the point of view of evolutionary theory. Gibert [20] adopted an evolutionary functional analysis of emotions to understand the basis of the difficulties in emotion processing experienced by people with SAD in social situations. From this standpoint, emotions are determined by the operation of three interlinked emotional systems. The most dominant system is the *threat and self-protection* system that seeks safety and avoidance of harm [21]. In social anxiety, fear is generated by social threats as well as physical ones. Thus, exclusion, rejection and being ignored deprive the person of desired social resources as well as safety. The other two systems connected with the threat system are the *drive seeking and acquisition* system associated with energising, exploring and obtaining resources for survival and the *affiliative, soothing and contentment* system which counters the sense of threat, promotes affiliation with others and allows the person to receive support from others. In social anxiety, the threat system is dominant, and this exaggerates social threats and fuels fears [22]. This dominance decreases the influence of the drive toward achievements. Moreover, it cannot be countered by the affiliative system as the attachment experiences of receiving care and support that develop this system and foster positive views of self and others are generally lacking in socially anxious people [8]. On the basis of negative early attachment experiences, as adults, socially anxious people most often display an anxious form of insecure attachment in which while they desire contact with others, they see themselves as unworthy and inadequate [23].

Due to the dominance of the threat and self-protection system, socially anxious people interpret their interactions with others in terms of a social hierarchy in which they locate themselves as at the lower end [20]. The goal of the socially anxious person then becomes to maintain a stable intermediate position in the social hierarchy [24]. FNE emphasises avoidance of moving downward in the hierarchy, which would ultimately lead to exclusion and loneliness. FPE prevents upward movements emanating from successes that garner the attention of people higher in the hierarchy and place them in conflict with those group members and make them subject to reprisals.

In the 14 years since the original proposal of FPE by Weeks [25], a considerable body of evidence has emerged to support the BFOE model. Building on a narrative review by Reichenberger and Blechert [24] and an initial systematic review by Fredrick and Luebbe [26], Cook et al. [27] conducted the first systematic review and meta-analysis on the role of FPE and FNE in social anxiety. Utilising data from 147 studies, their findings confirmed FNE and FPE as distinct constructs with similarly strong associations with SA. Meta-correlations showed that the relationship of FPE and FNE to SA was similar, but FPE and FNE were only moderately correlated (*r* = 0.48). Cook et al. confirmed the importance of FNE and FPE in social anxiety in two ways. First, people meeting criteria for a diagnosis of SAD scored significantly higher on both FNE and FPE than individuals not diagnosed with SAD. Second, FNE and FPE accounted for 42% of the variance in overall SA across a range of measures of SA. In dividing the variance accounted for by FNE and FPE, 54.8% of the variance was shared, 11% unique to FNE and 8% unique to FPE. Thus, FPE and FNE are elevated in people with SAD and are core features of social anxiety that act in combination to determine the strength of social anxiety.

### 1.2. The Extended Bivalent Fear of Evaluation Model (Cook et al., [15])

As an elaboration of the BFOE framework, Cook et al. [15] proposed a model which added two further forms of cognitive distortion: concerns of social reprisal (CSRs) and disqualification of positive social outcomes (DPSOS). These cognitive distortions are also consistent with evolutionary explanations of social anxiety. Concerns about social reprisal capture intense fears of the aggressive actions that may follow from the evaluations made by others. These concerns are linked to FNE given that these fears are based on concerns that others will appraise the person negatively and respond punitively to any evidence of personal flaws or social failures. On the other hand, concerns about reprisal are linked to FPE because positive performances make the person more prominent in the group and are more likely to result in the person being seen as a rival and a threat to others higher up in the hierarchy. Discounting positive social outcomes is a means of reducing attention to oneself and constitutes a submissive response to others higher in the group that placates potential criticism or rivalry. This involves minimally attributing social success to one’s own efforts or attributing the success to external factors such as the benevolence of others or luck [15].

In their research, Cook et al. [15] found evidence for a model including FNE, FPE, CSR and DPSOS. Structural equation modelling demonstrated that FNE and FPE significantly predicted social anxiety and their effects were partially mediated by CSR and DPSOS. There was evidence of serial mediation such that higher levels of FNE and FPE predicted higher levels of CSR, which in turn predicted higher levels of DPSOS and social anxiety. Notably, although both were significant in the model, FNE was a stronger predictor of social anxiety than FPE. In Cook et al. [15], social anxiety was operationalised by the Social Phobia scale (SPS; [28]), which focuses on social situations in which the person feels conspicuous to others (e.g., when eating in front of others) or fears their anxiety symptoms are apparent to others (e.g., fears of blushing).

Cook et al. [15] developed their model with an Australian sample, i.e., from an essentially Western country. However, to date, the only replication study has been conducted by Okawa et al. [29] with a Japanese sample, an essentially Eastern country. Okawa et al.’s structural equation modelling identified the same pattern of relationships in their Japanese sample. Once more, while FNE was a stronger predictor of social anxiety than FPE, both forms of evaluative fear significantly predicted social anxiety, and both were partially serially mediated by CRS and DPSOS. In the Okawa et al. study, social anxiety was operationalised with the Social Interaction Anxiety Scale (SIAS [28]). Despite having a moderate to strong correlation with SPS (*r* = 0.70), SIAS focuses on different aspects of social anxiety that capture subjective discomfort when interacting with others (e.g., feeling uncomfortable or tense when talking in a group or with an individual). Okawa et al. [29] concluded that while the relationship between FNE and FPE was not as strong in their Japanese sample compared to Cook et al. (*r* = 0.18 vs. *r* = 0.51), Cook et al.’s model appeared to apply in collectivist as well as more individualistic cultures.

### 1.3. The Present Study

The aim of this study was to replicate and extend the findings of Cook et al. [15] and Okawa et al. [29], who found evidence that the prediction of social anxiety by FNE and FPE is partially and serially mediated by cognitive distortions related to fears of reprisal and discounting positive outcomes. We utilised an Australian sample and extended these studies in two ways. First, we combined both measures of social anxiety (the Social Phobia scale and the Social Interaction Anxiety Scale) used separately in previous research. This extended the previous studies by providing a broader and more representative coverage of social anxiety symptoms related to performance and social interaction. Second, we tested how the model applied to participants identified with probable SAD relative to participants without SAD to explore any possible differences between respondents based on the severity of their symptoms of social anxiety.

## 2. Materials and Methods

### 2.1. Participants and Procedure

The sample comprised eight hundred and ninety university students with a mean age of 29.49 years (*SD* 9.84 years). As shown in Table 1, the majority were female (74.5%), had a partner (64.6%) and had a certificate or above qualification (62.2%). Notably, 20.7% had an undergraduate or post-graduate degree and four people had not completed high school. These people were students in an open access degree program which provides an online study option for people wishing to study a second degree, to switch careers or who have not had the opportunity to study at university, most often due to work or family commitments. Based on the Social Phobia Scale (SPS [28]), 63.7% did not meet the cut-off for probable SAD (63.7%). The participants were recruited from a university undergraduate program where participation was associated with course credit. They completed an online survey anonymously, giving implicit consent by submission of the survey, which took approximately 60 min to complete. The study was approved by the Swinburne University of technology ethics committee. The ethical clearance did not permit collection of data about the students’ mental health diagnoses, treatment experiences or medication histories.

### 2.2. Measures

#### 2.2.1. Social Anxiety

The ***Social Phobia Scale*** (SPS; Ref. [28]) is a 20-item scale of social anxiety experienced in performance-related situation symptoms. According to [30], it measures *fears of overt evaluation* (e.g., when in an elevator, I am tense if people look at me) and *fears of attracting attention* (e.g., I worry I might do something to attract the attention of others). SPS items are rated on a five-point rating scale (0 = “not at all”–4 = “extremely”), with summed higher scores representing greater social anxiety. A total SPS score of greater or equal to 24 has been established for use as a clinical cut-off score to distinguish those with probable SAD from respondents without SAD [31]. The scale shows excellent internal consistency (alpha = 0.95) and convergent and discriminant validity [28]. In the present study, internal consistency was excellent (alpha = 0.95).

The ***Social Interaction Anxiety Scale*** (SIAS, Ref. [28]) was developed as a complementary measure to the SPS which focuses on internal discomfort in social interactions such as individual or group conversations. The full scale includes 20 items rated on a five-point Likert scale ranging from 0 (not at all characteristic or true of me) to 4 (extremely characteristic or true of me). In this study, however, we used a short form of the SIAS developed by [30]. The SIAS-S measure comprised five items: “When mixing socially I am uncomfortable”, “I have difficulty talking with other people”, “I find myself worrying that I won’t know what to say in social situations”, “I am nervous mixing with people I don’t know well” and “I am tense mixing in a group”. In this study, these items showed excellent internal consistency (alpha = 0.95).

#### 2.2.2. BFOE Measures

The ***Brief Fear of Negative Evaluation Scale—Straightforward*** (BFNE-S; Ref. [32]) is a 12-item scale that measures fear of negative evaluation (e.g., “I often worry that I will say or do wrong things”). BFNE-S items are rated on a five-point rating scale (1 = “not at all characteristic of me”–5 = “extremely characteristic of me”), with summed higher scores indicating greater FNE. The straightforward version (omitting negatively scored items) was used as these items have been found to be more reliable and valid indicators of FNE in undergraduate and clinical samples [17,32]. The BFNE-S also shows excellent reliability and factorial and construct validity [32]. In this study, internal consistency was excellent (alpha = 0.97).

The ***Fear of Positive Evaluation Scale*** (FPES; Ref. [25]) is an 8-item scale that measures fear of positive evaluation (e.g., “I generally feel uncomfortable when people give me compliments”). FPES items are rated on a ten-point rating scale (from 0 = “not at all true” to 9 = “very true”), with summed higher scores representing greater FPE. Respondents make their ratings in relation to someone they do not know very well to overcome the influence of familiarity, which can reduce social anxiety [33]. Weeks et al. [25] confirmed the factorial validity of the FPES and reported good internal consistency (alpha = 0.80) and test–retest reliability (Intraclass correlation co-efficient = 0.70, *p* < 0.001). The FPES also showed sound convergent validity with BFNE-S and the SIAS-S and divergent validity with measures of depression, worry and generalised anxiety [34]. In this study, internal consistency was very good (alpha = 0.80).

The ***Concerns of Social Reprisal Scale*** (CSRS; Ref. [35]) is a 10-item scale that measures concerns of social reprisal (e.g., “I could see making a good impression on others as being threatening to some people”). CSRS items are rated on a ten-point rating scale (0 = “not at all true”–9 = “very true”), with summed higher scores representing greater concerns. Like the FPE measure, respondents make their ratings in relation to someone they do not know very well to overcome the influence of familiarity. Weeks et al. [35] reported good internal reliability (alpha = 0.85) and Cook et al. [15] reported excellent internal consistency (alpha = 0.95). In addition, Weeks et al. [35] reported good convergent and discriminant validity for the CSRS. In this study, internal consistency was excellent (alpha = 0.92).

The ***Disqualifications of Positive Social Outcomes Scale*** (DPSOS; Ref. [36] is an 11-item scale that measures disqualifications of positive social outcomes (e.g., “I frequently dismiss my own social successes and accomplishments”). DPSOS items are rated on a ten-point rating scale (1 = “not at all true”–10 = “very true”), with summed higher scores representing greater disqualification. Following Cook et al. [15], the DPSOS was treated as one scale. The DPSOS has shown excellent internal consistency (alpha = 0.94 [15]) and good construct validity [36]. In this study, internal consistency was excellent (alpha = 0.94).

### 2.3. Data Analysis Strategy

The response format did not permit progression in the online survey unless all questions had been answered. Consequently, there were no missing data. Data were checked for miscoded values. Structural equation modelling assumptions regarding sample size, multivariate normality, linearity, multicollinearity and directionality were checked. Social anxiety was measured as a latent variable comprising the SPS and SIAS measures of social anxiety. The sample size was guided by the Bentler and Chou [37] recommendation of a minimum of 5–10 cases per free parameter. The paths were specified based on the research hypothesis and modification indices. The model fit was evaluated using Kline’s [38] recommendation of a Chi-square test accompanied by the following fit indices: Comparative Fit Index (CFI; Bentler [39]), The Tucker–Lewis Index (TLI; Ref. [40]) Steiger–Lind Root Mean Squared Error of Approximation (RMSEA; Ref. [41]) and Standardised Root Mean Square Residual (SRMR; [38]). Acceptable cut-off values suggested by Hu and Bentler [42]) were applied; CFI ≥ 0.95, TLI ≥ 0.95, RMSEA ≤ 0.08 and SRMR ≤ 0.06. To control for common method variance and demonstrate the independence and discriminant validity of the items of the SPS and SIAS-S, these and all the other scale items were checked using the heterotrait–monotrait (HTMT) ratio of correlations [43] where a value less than 0.90 indicates the discriminate validity between the factors. Data analysis was performed using SPSS v29, AMOS v29 and R 4.2.1.

## 3. Results

### 3.1. Data Screening

Prior to undertaking SEM, an MANOVA was conducted to see the effects of categorical demographic variables of gender employment status, SA category and education level on the study variables. Gender, education level and employment status revealed no statistically significant differences across all study variables (all *p*’s > 0.05). Statistically significant differences between SA categories were evident for all variables; however, these effects were small to moderate, with the largest being for SPS (η^2^ = 0.65). Age was negatively correlated with all study variables, suggesting that younger participants tended to report greater FPE, FNE, DPSOS, CSRS, SPS and SIAS-S anxiety but the strength of the relationship was very weak. When age was controlled in the SEM, the paths and their significance remained the same; given this, age was not included in the final model.

### 3.2. Correlational Analyses

Pearson’s product correlational analyses indicated significant positive correlation among all variables (see Table 2). Thus, higher social anxiety symptoms (SPS and SIAS-S) were associated with greater FNE and FPE, more CSRS, and greater DPSOS. Notably, FNE and FPE were moderately correlated (*r* = 0.54), which is consistent with other research showing they are overlapping but distinct constructs [27]. HTMT analysis indicated independence and discriminant validity across the study variables ranging between 0.794 (SPS and SIAS-S) and 0.481 (SIAS-S and CSRS), confirming discriminant validity amongst the scales.

### 3.3. Testing the Theoretical Model

**Figure 1:**
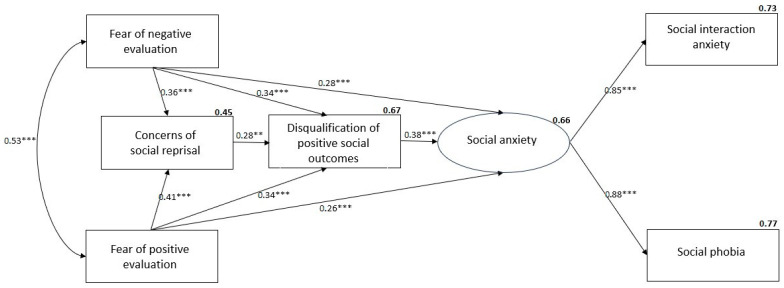
SEM of prediction of social anxiety with beta weights and squared multiple correlations (*** *p* < 0.001, ** *p* < 0.01).

The hypothesised model, shown in Figure 1, described the data reasonably well (*χ*^2^(4) = 9.57, CFI = 0.990, TLI = 0.961, RMSEA = 0.075, SRMR = 0.014), explained 77% of the variation in SPS and 73% of the variation in SIAS-S. A review of the SEM pathways indicated that, as expected, FNE had a significant direct influence on CSRS (*β* = 0.36, *p* < 0.001), and DPSOS (*β* = 0.34, *p* < 0.001). DPSOS was found to mediate the relationships between FNE and CSRS with social anxiety. FPE also had a significant direct influence on CSRS (*β* = 0.41, *p* < 0.001), and DPSOS (*β* = 0.34, *p* < 0.001). Thus, FNE and FPE directly predict higher levels of both forms of social anxiety and also have an indirect effect on social anxiety through their prediction of higher levels of fear of reprisal and of the tendency to discount positive social experiences. An invariance analysis indicated that overall, the strength of the pathways across the model did not differ significantly across gender (∆*χ*^2^(1) = 3.18, *p* = 0.074, ∆CFI = 0.011, ∆TLI = 0.002, RMSEA = 0.002) and so the same patterns of relationships existed for men and women.

### 3.4. Model Comparison for Probable with and Without SAD Groups

**Figure 2:**
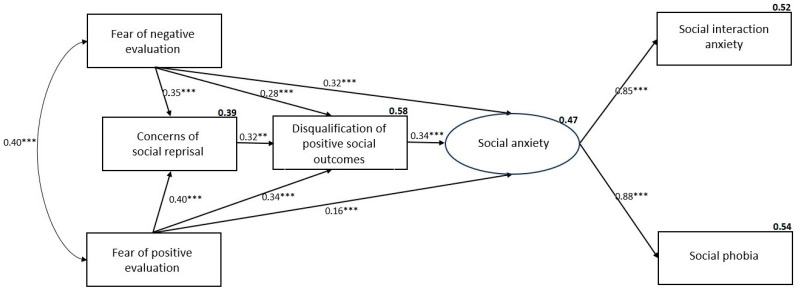
Model for participants with probable SAD with beta weights and squared multiple correlations (*** *p* < 0.001, ** *p* < 0.01).

**Figure 3:**
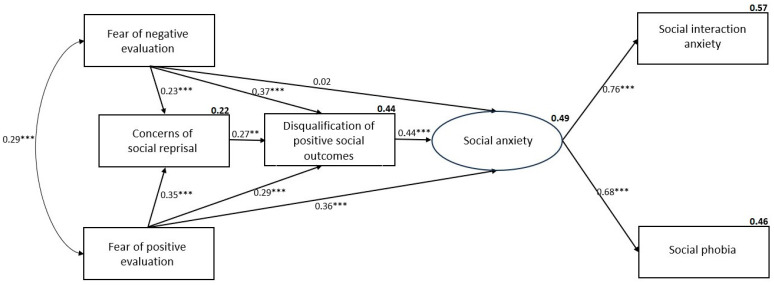
Model for participants without probable SAD with beta weights and squared multiple correlations (*** *p* < 0.001, ** *p* < 0.01).

To explore potential group differences across the severity of social anxiety, the model was tested for those with or without probable SAD. Results indicated that the model was valid across both categories. Specifically, the model using the probable SAD sample had a good fit (*χ*^2^(4) = 6.61, CFI = 0.983, TLI = 0.935, RMSEA = 0.070, SRMR = 0.023), accounting for 54% of the variation in SPS and 52% of the variation in SIAS-S (see Figure 2). Similarly, the model using the without SAD sample had a good fit (*χ*^2^(5) = 2.57,CFI = 0.984, TLI = 0.953, RMSEA = 0.072, SRMR = 0.027), accounting for 46% of the variation in SPS and 57% of the variation in SIAS-S (see Figure 3). The only substantial difference between the models and the overall model was that the direct pathway for FNE was non-significant in the group without probable SAD all other pathways were significant as in the other models.

### 3.5. Multigroup Comparisons

A multigroup comparison was conducted using confirmatory factor analysis to determine whether the final model differed between the group with probable SAD in comparison to those without probable SAD. The Z-test for the equality of structural coefficients across the multiple groups revealed significant differences between disqualification of positive social outcomes and SA (Z = 2.83; *p* = 0.002) and between FPE and SA (Z = 3.57; *p* < 0.001). This indicates that disqualification of positive social outcomes and FPE had a stronger relationship with social anxiety symptoms for people without probable SAD compared to people with probable SAD. Additionally, significant differences were found for FNE and SA (*Z* = 2.16; *p* = 0.015), indicating that FNE had a stronger relationship with social anxiety symptoms for people with probable SAD compared to people without probable SAD. Table 3 presents the standardised weights for both models.

## 4. Discussion

The purpose of this study was to replicate and extend the previous studies of Cook et al. [15] and Okawa et al. [29] by incorporating a more general assessment of social anxiety and determining whether the extended BFOE model applies equally to people with probable SAD and those without probable SAD. Consistent with the previous findings, our analyses showed that the extended model was a good fit for our overall sample and for the subgroup of people with probable SAD. The model was also a good fit for people without SAD with the exception that the direct predictive pathway for FNE was no longer significant. Thus, for those low on social anxiety, the influence of FNE was indirect and determined by the cognitive processes of fears of reprisal and disqualification of positive social outcomes. Our discussion focuses on the implications of these findings for the extended BFOE model of social anxiety and directions for future research.

Our findings provide further confirmation of the BFOE model in that FNE and FPE were moderately correlated but made independent contributions to the prediction of social anxiety. This supports the contention that FPE and FNE are related but distinct constructs. That the group of participants with probable SAD scored higher on FNE and FPE as well as concerns about reprisal and discounting positive social outcomes underlines the importance of these variables in social anxiety and further supports the findings of past research reviewed by Cook et al. [27]. One difference in our data was that FNE and FPE had approximately equivalent relationships with both measures of social anxiety. Cook et al. [27] concluded that across all the studies reviewed, FNE tends to have a stronger relationship with social interaction anxiety (e.g., SIAS) and FPE a stronger relationship with social performance anxiety (e.g., SPS). However, Cook et al. only identified this as a weak tendency that varies across studies, so this does not qualify our overall findings.

The different direct contribution of FNE to the models for those with and without probable SAD is an interesting finding. In the overall model, the weightings for FNE and FPE were approximately equal (FNE β = 0.28; FPE β = 0.26). However, for the group with probable PTSD, the weight increased for FNE (β = 0.32) but decreased for FPE (β = 0.16) and multigroup comparisons suggested that the direct effect for FNE was significantly higher in this group than it was in the non-SAD group where the FNE weight was negligible (β = 0.02). Further, multigroup comparisons for FPE showed a significantly lower contribution to predicting social anxiety in the probable SAD group compared to the non-SAD group (β = 0.16 vs. =0.36). The prominence of FNE as a direct predictor in the probable SAD group is consistent with the emphasis given to FNE in DSM-5-TR. That FPE remained a significant, albeit weaker, direct predictor in the probable SAD group suggests that the direct contribution of FPE is also important in this group. That concerns about reprisal and disqualification of positive social outcomes provide significant indirect pathways supports Weeks call for interventions to include these variables as treatment targets alongside FNE [44].

That FNE was no longer a direct predictor in the non-SAD group is an intriguing finding. This suggests that while social anxiety can generally be assumed to be on a continuum of severity [2], there may be a qualitative shift in the intensity and disruptive effect of FNE at clinical levels of social anxiety compared to at lower levels of social anxiety. This also reinforces the importance of FPE (along with the mediating variables) in the determination of social anxiety at all levels of distress. It is also possible that the direct effect of FNE is determined by other factors not included in the extended BFOE model

Gilbert’s psychoevolutionary model of SAD [20,21,22] offers some possible variables that may have an impact on the influence of FNE in the extended BFOE system. This BFOE model confines itself to measures of constructs that tap the influence of the threat and safety system within Gilbert’s framework. This is understandable given that the threat and safety system is the most prominent one in human life and is dominant in social anxiety [20]. What has yet to be operationalised and explored, however, is the role in this process of the other motivational systems of competitiveness and acquisition and affiliation proposed by Gilbert [20,22]. The affiliative system may be of particular importance as, theoretically, it is attributed a crucial role in deactivating and countering the threat and safety system and may act as a moderator of the threat related components of the extended bivalent model.

According to Gilbert [22], the threat system that generates FNE and FPE involves threat detection and initiates responses designed to eliminate harm and seek safety. In social anxiety, safety seeking behaviours are linked to monitoring for social threats and are directed at avoiding any actions in a social encounter that might lead to rejection and exclusion. In contrast to the threat and safety system, the affiliative system operates according to a sense of safeness rather than safety. Safeness is not connected to avoidance of threat but instead seeks out safe and supportive internal and external resources that support coping with threats and encourage social exploration. A person operating from an affiliative motivation seeks to enjoy social interactions with others, downplay perceived social threats and activate non-competitive affiliative behaviours. Gilbert [22] identified self-assurance (remembering past successes and drawing upon learnt skills) and self-compassion (benevolent self-responding) as examples of internal processes linked to the generation of social safeness. In support of this, research shows that self-compassionate self-responding, particularly reducing uncompassionate behaviours such as self-criticism, directly predicts lower levels of social anxiety [14,45].

The theoretical possibility that the affiliative system can influence the operation of FNE and FPE within the bivalent model offers a direction for further elaboration and research with the model. Including existing measures of affiliation processes such as general social safeness [46] and self-compassion [47] would allow a more comprehensive appraisal of the determinants of social anxiety. It would also provide a test of the possibility that social safeness or self-compassion may moderate the effect of FNE and FPE such that the influence of these variables on social anxiety is attenuated in people high in social safeness and self-compassion.

Our findings also have implications for working clinically with people experiencing SAD. Although cognitive behavioural approaches have become the gold standard for interventions in SAD, there remains a substantial number of clients who do not respond to cognitive behavioural treatment or are left with residual symptoms after treatment [8,9]. There is a need, therefore, to further refine these methods to ensure that all important features of SAD are addressed. On the basis of our findings and earlier studies on the BFOE [15,29], evaluation fears are clearly not confined to FNE; FPE is clearly important in SAD. Yet, to date, only one study has trialled the incorporation of specific intervention strategies to address FPE [44]. Similarly, while acknowledged as a feature of the thinking of socially anxious people, the tendency to disqualify positive social experiences and fears of reprisal are not routinely targeted directly in SAD treatment [35,36]. As these two processes are significant mediators of the effects of FNE and FPE, specific interventions to help clients identify the operation of these processes and strategies to reduce their effects will play an important role in treatment and prevention of SAD.

### Limitations and Future Directions

Our study is not without limitations. Although our sample of 890 participants was considerably larger than the two previous studies which tested the full model (Cook et al. [15], *n* = 255; Okawa et al. [29], *n* = 496), it was similarly biassed towards inclusion of female participants and confined to an undergraduate student sample. Moreover, while we identified a group of respondents with probable SAD based on the SPS cut-off for clinical levels of social anxiety, we did not assess a clinical sample of participants. That the SPS was also one of our measures of social anxiety is a further limitation. Future research is needed to assess the generalisability of the model with a more balanced gender distribution and in samples of people diagnosed with SAD, preferably by clinical interview (e.g., the structured clinical interview for DSM-5 [48]). Interestingly, studies that have compared diagnosed SAD participants with those with probable SAD matched for gender have often found equivalence. For example, McBride et al. [45] found no differences between an SAD group and a probable group regarding levels of social anxiety symptoms and emotion regulation deficits and both groups were significantly higher on these variables than a non-SAD group. Indeed, the probable SAD group was significantly higher on self-directed uncompassionate behaviour, specifically self-criticism, than the clinical SAD group.

As with the previous studies on the extended BFOE model [15,29], our research is limited to cross-sectional assessment of the predictive relationships in the SEM model and we also have not controlled for the influence of comorbid conditions such as depression, which is closely related to SAD [8]. Future work is needed to establish longitudinal relationships and to control for the effects of comorbid conditions such as depression and anxiety as covariates in the SEM model. Network analysis may also be an alternate approach to SEM to explore complex constructs that may not fit neatly into predefined factors that were evaluated in the current study [49]. Future work could also examine the effects of treatment interventions for SAD in clinical samples on the variables in the model.

There is also a need to further clarify the measurement of FNE and FPE. Whereas FPE appears to be a unique predictor of social anxiety, FNE is also a predictor of depression, albeit at a lower magnitude [19]. This suggests that the current FNE measurement of FNE may include generalised aspects of evaluative fears that relate to other emotions. Similarly, research on the FPE measure suggests that it may capture aspects of evaluative fear that are not specific to FPE. Wilson et al. [50] asked an SAD group and a non-SAD comparison group to explain the reasons for their ratings of the eight straightforward items of the FPE. They found that, consistent with the BFOE theory, the responses primarily reflected fears of proximal or eventual negative consequences of positive social evaluations and the SAD group endorsed these reasons to a greater extent than did the non-SAD comparison group. However, several other reasons were given which did not reflect such fears, such as heightened self-consciousness and uncertainty about how to respond to the positive feedback. Thus, FPE seems to overlap with other constructs relevant to SAD and may be mediated by those factors.

In response to concerns about measurement of evaluative fears, Weeks et al. [51] have recently factor analysed a combination of the FNE and FPE scales. In their new measure, they created more focused subscale measures of FNE and FPE and identified a third factor they defined as an intervalent factor which captures aspects of evaluation that are not specifically negative or positive (e.g., I worry about what people will think of me even when I know it does not make any difference). In line with the more generalised FNE construct, four of the five items on the intervalent scale came from the FNE measure and one from the FPE measure. Weeks et al. found that all three scales related more strongly to social anxiety measures than measures of depression, suggesting good discriminant validity of the new measure. The establishment of factorial validity for the three dimensions also addresses the concerns over the degree of overlapping content in the two measures [51].

## 5. Conclusions

In conclusion, the findings from this study provide strong support for the extended bivalent mode of fear of evaluation. We extended previous analyses by showing that the model applies to both social performance and interaction anxiety and is a good fit for people with and without probable SAD. These findings reinforce calls for FPE to more directly addressed in the treatment of social anxiety [44]. The data also raise the possible extension of the model to incorporate the possible moderating influence of the affiliative system of evaluative fear and the need to account for the effects of comorbid conditions such as depression.

## Figures and Tables

**Table 1:** Participants’ demographic information.

Demographic	*n*	%
*Gender*		
Male	218	24.5%
Female	666	74.8%
No answer	6	0.7%
*Relationship status*		
Single	325	36.5%
Partnered	144	16.2%
Partnered and living together	187	21.0%
Married	201	22.6%
Separated	25	2.8%
Other	8	0.9%
*Educational level*		
Below high school	4	0.4%
High school	332	37.3%
Certificate, diploma or trade	369	41.5%
Undergraduate degree	149	16.7%
Postgraduate degree	36	4.0%
*Employment status*		
Unemployed	176	19.8%
Casual or part-time employed	353	39.7%
Full-time employed	283	31.8%
Homemaker	64	7.2%
Retired or unable to work	14	1.6%
*Country of birth*		
Australia	718	80.7%
Asia	23	2.6%
United Kingdom	20	2.2%
Europe (excluding UK)	12	1.3%
New Zealand	19	2.1%
South Africa	11	1.2%
Other	16	9.8%
*Probable SAD*		
No	567	63.7%
Yes	323	36.3%

Note. *N* = 890. Probable SAD calculated as a score of ≥24 on the Social Phobia Scale.

**Table 2:** Correlations, internal consistencies (Cronbach alpha), scale means and standard deviations of study variables (N = 890).

Variables	1	2	3	4	5	Mean (SD)	α
1. FPES	–	–	–	–		26.96 (17.67)	0.80
2. BFNE-S	0.54 ***	–	–	–		20.95 (9.35)	0.97
3. DPSOS	0.70 ***	0.69 ***	–	–		32.82 (23.05)	0.94
4. CSRS	0.60 ***	0.58 ***	0.69 ***	–		27.73 (19.18)	0.92
5. SPS	0.59 ***	0.63 ***	0.65 ***	0.51 ***		16.02 (14.95)	0.95
6. SIAS-S	0.59 ***	0.55 ***	0.66 ***	0.45 ***	0.89 ***	6.85 (5.80)	0.95

Note. FPES = Fear of Positive Evaluation Scale; BFNE-S = Brief Fear of Negative Evaluation Scale—Straightforward; DPSOS = Disqualification of Positive Social Outcomes Scale; CSRS = Concerns of Social Reprisal Scale; SPS = Social Phobia Scale; SIAS-S = Social Interaction Anxiety Scale. (*** *p* < 0.001).

**Table 3:** Standard regression weights estimates for multi-group analysis.

Path Direction	Beta Weights		
	No SAD	SAD	Z Value and *p*-Value
BFNE-S→CSRS	0.23	0.35	Z = 0.67, *p* = 0.749
FPES→CSRS	0.35	0.40	Z = 0.06, *p* = 0.524
BFNE-S→DPSOS	0.32	0.28	Z = 1.28, *p* = 0.900
FPES→DPSOS	0.29	0.34	Z = 1.07, *p* = 0.858
CSRS→DPSOS	0.27	0.32	Z = 1.71, *p* = 0.956
DPSOS→SA	0.44	0.34	Z = 2.83, *p* = 0.002 **
BFNE-S→SA	0.02	0.31	Z = 2.16, *p* = 0.015 *
FPES→SA	0.36	0.16	Z = 3.57, *p* < 0.001 ***

Note. FPES = Fear of Positive Evaluation Scale; BFNE-S = Brief Fear of Negative Evaluation Scale—Straightforward; DPSOS = Disqualification of Positive Social Outcomes Scale; CSRS = Concerns of Social Reprisal Scale; SA-Social Anxiety. (*** *p* < 0.001, ** *p* < 0.01, * *p* < 0.05).

## Data Availability

The data supporting the conclusions of this article can be provided by the corresponding author upon reasonable request and university ethics approval.
